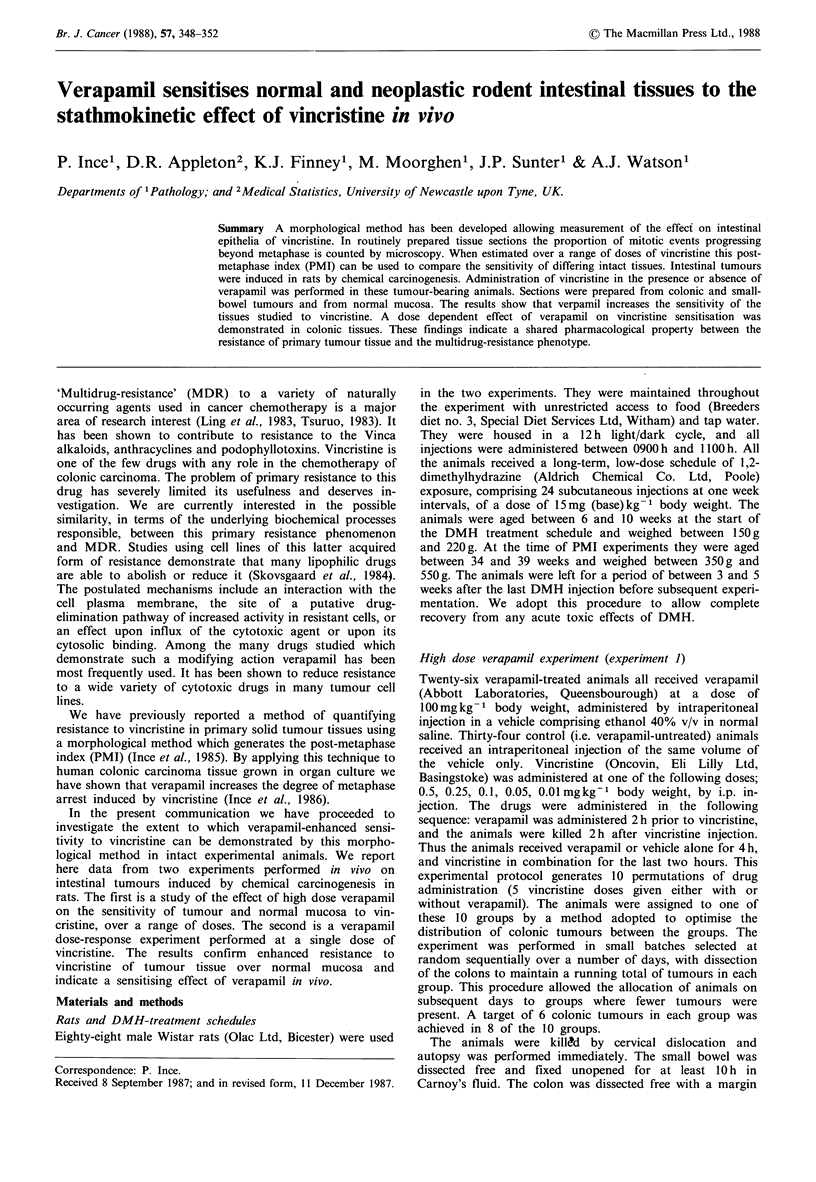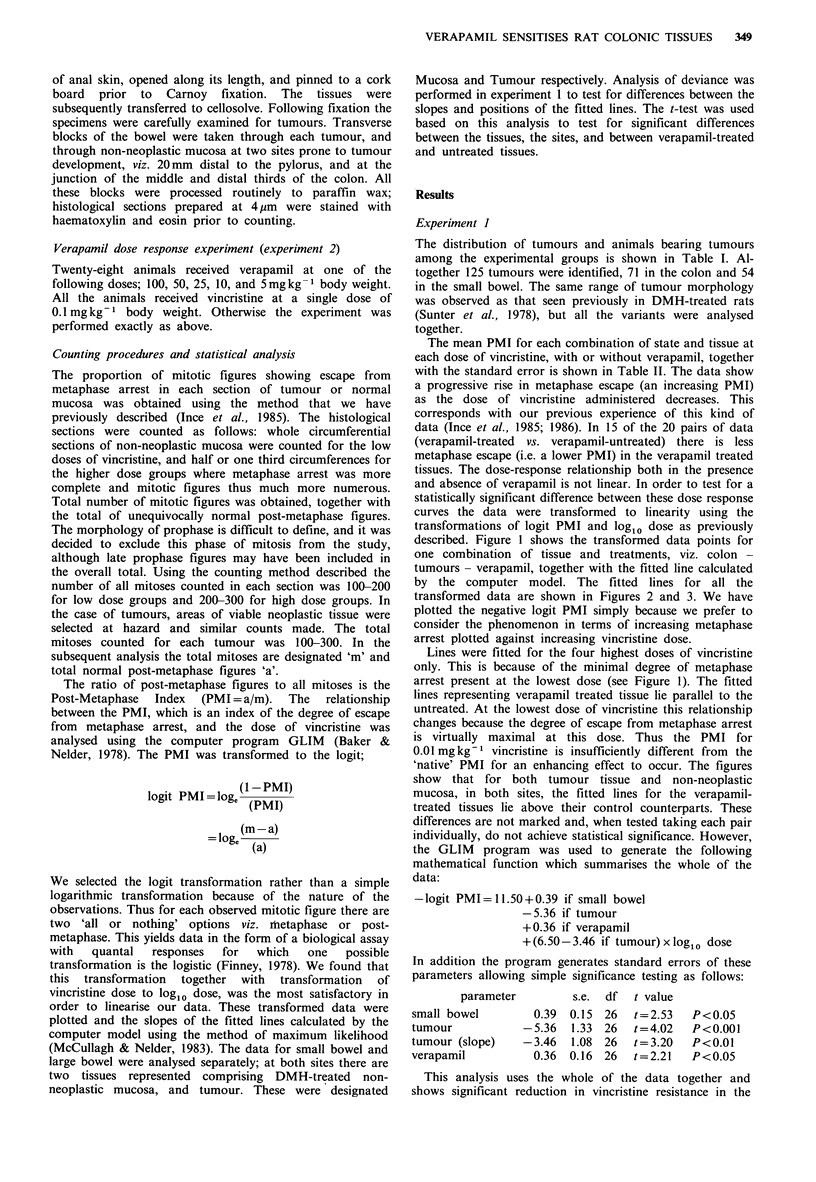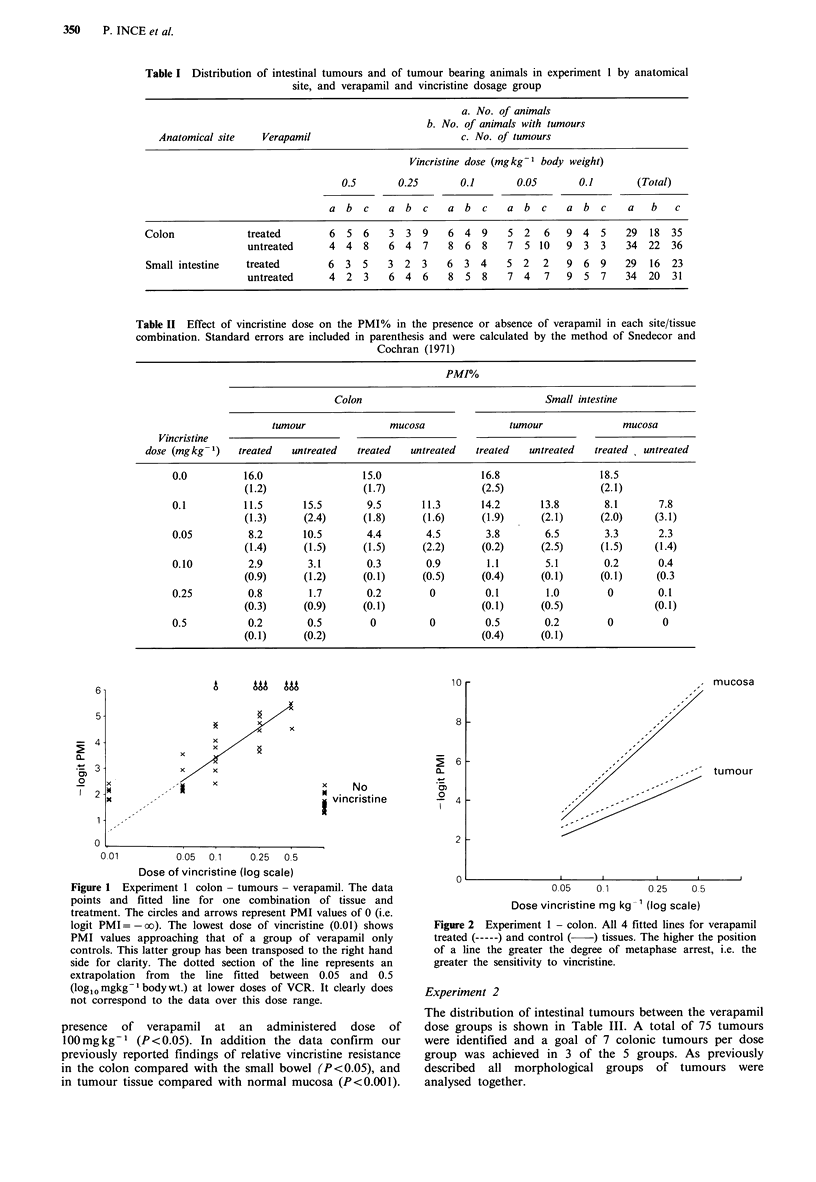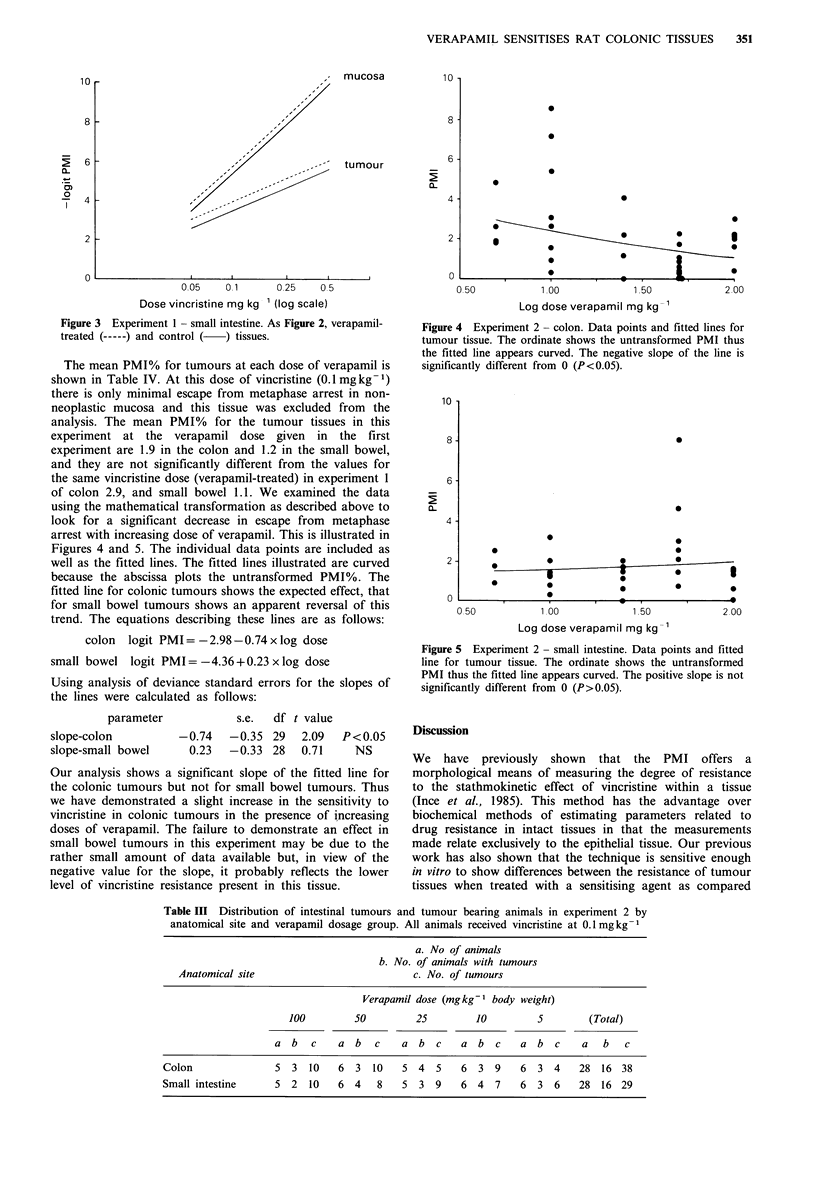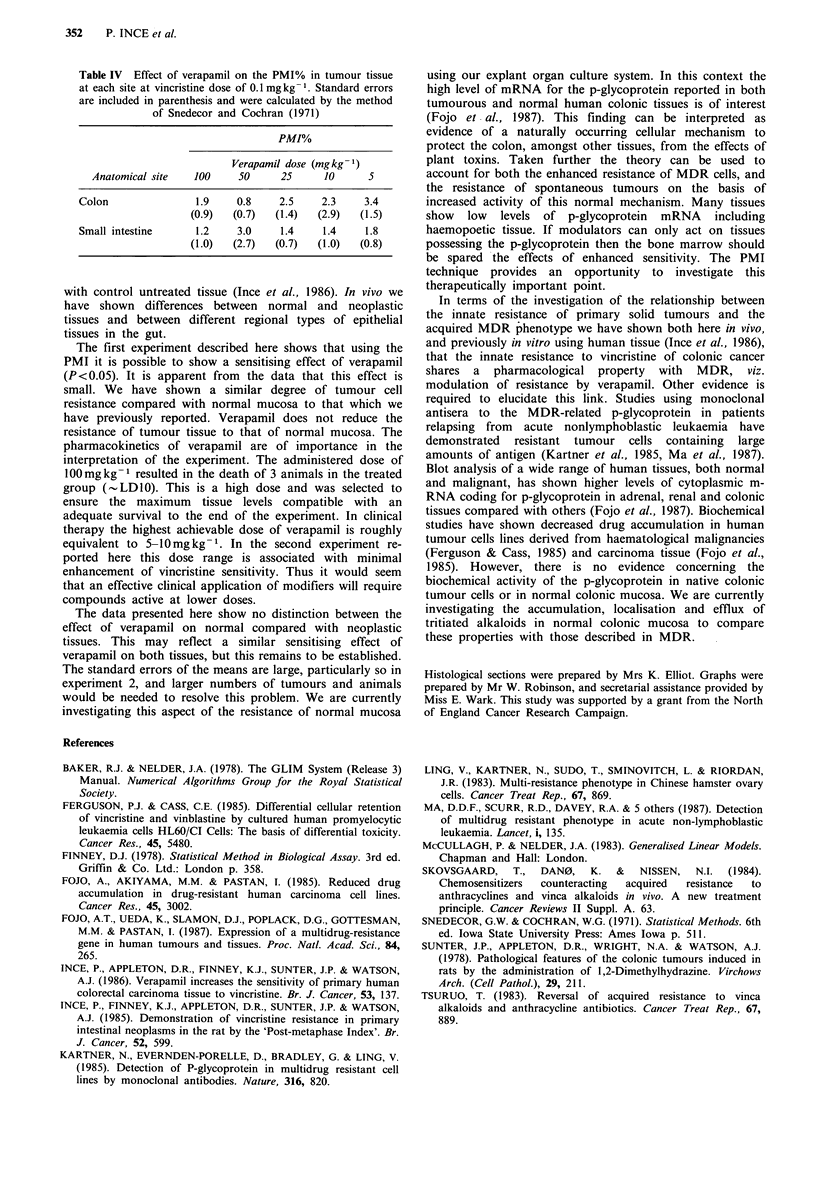# Verapamil sensitizes normal and neoplastic rodent intestinal tissues to the stathmokinetic effect of vincristine in vivo.

**DOI:** 10.1038/bjc.1988.80

**Published:** 1988-04

**Authors:** P. Ince, D. R. Appleton, K. J. Finney, M. Moorghen, J. P. Sunter, A. J. Watson

**Affiliations:** Department of Pathology, University of Newcastle upon Tyne, UK.

## Abstract

A morphological method has been developed allowing measurement of the effect on intestinal epithelia of vincristine. In routinely prepared tissue sections the proportion of mitotic events progressing beyond metaphase is counted by microscopy. When estimated over a range of doses of vincristine this post-metaphase index (PMI) can be used to compare the sensitivity of differing intact tissues. Intestinal tumours were induced in rats by chemical carcinogenesis. Administration of vincristine in the presence or absence of verapamil was performed in these tumour-bearing animals. Sections were prepared from colonic and small-bowel tumours and from normal mucosa. The results show that verapamil increases the sensitivity of the tissues studied to vincristine. A dose dependent effect of verapamil on vincristine sensitisation was demonstrated in colonic tissues. These findings indicate a shared pharmacological property between the resistance of primary tumour tissue and the multidrug-resistance phenotype.


Br. J. Cancer (1988), 57, 348-352 © The Macmillan Press Ltd., 1988

### Verapamil sensitises normal and neoplastic rodent intestinal tissues to the stathmokinetic effect of vincristine *in vivo*

P. Ince[1], D.R. Appleton[2], K.J. Finney[1], M. Moorghen[1], J.P. Sunter[1] & A.J. Watson[1]

*Departments of [1]Pathology; and [2]Medical Statistics, University of Newcastle upon Tyne, UK.*

**Summary** A morphological method has been developed allowing measurement of the effect on intestinal epithelia of vincristine. In routinely prepared tissue sections the proportion of mitotic events progressing beyond metaphase is counted by microscopy. When estimated over a range of doses of vincristine this post-metaphase index (PMI) can be used to compare the sensitivity of differing intact tissues. Intestinal tumours were induced in rats by chemical carcinogenesis. Administration of vincristine in the presence or absence of verapamil was performed in these tumour-bearing animals. Sections were prepared from colonic and small-bowel tumours and from normal mucosa. The results show that verpamil increases the sensitivity of the tissues studied to vincristine. A dose dependent effect of verapamil on vincristine sensitisation was demonstrated in colonic tissues. These findings indicate a shared pharmacological property between the resistance of primary tumour tissue and the multidrug-resistance phenotype.

'Multidrug-resistance' (MDR) to a variety of naturally occurring agents used in cancer chemotherapy is a major area of research interest (Ling *et al.*, 1983, Tsuruo, 1983). It has been shown to contribute to resistance to the Vinca alkaloids, anthracyclines and podophyllotoxins. Vincristine is one of the few drugs with any role in the chemotherapy of colonic carcinoma. The problem of primary resistance to this drug has severely limited its usefulness and deserves investigation. We are currently interested in the possible similarity, in terms of the underlying biochemical processes responsible, between this primary resistance phenomenon and MDR. Studies using cell lines of this latter acquired form of resistance demonstrate that many lipophilic drugs are able to abolish or reduce it (Skovsgaard *et al.*, 1984). The postulated mechanisms include an interaction with the cell plasma membrane, the site of a putative drug-elimination pathway of increased activity in resistant cells, or an effect upon influx of the cytotoxic agent or upon its cytosolic binding. Among the many drugs studied which demonstrate such a modifying action verapamil has been most frequently used. It has been shown to reduce resistance to a wide variety of cytotoxic drugs in many tumour cell lines.

We have previously reported a method of quantifying resistance to vincristine in primary solid tumour tissues using a morphological method which generates the post-metaphase index (PMI) (Ince *et al.*, 1985). By applying this technique to human colonic carcinoma tissue grown in organ culture we have shown that verapamil increases the degree of metaphase arrest induced by vincristine (Ince *et al.*, 1986).

In the present communication we have proceeded to investigate the extent to which verapamil-enhanced sensitivity to vincristine can be demonstrated by this morphological method in intact experimental animals. We report here data from two experiments performed *in vivo* on intestinal tumours induced by chemical carcinogenesis in rats. The first is a study of the effect of high dose verapamil on the sensitivity of tumour and normal mucosa to vincristine, over a range of doses. The second is a verapamil dose-response experiment performed at a single dose of vincristine. The results confirm enhanced resistance to vincristine of tumour tissue over normal mucosa and indicate a sensitising effect of verapamil *in vivo*.

### Materials and methods

### *Rats and DMH-treatment schedules*

Eighty-eight male Wistar rats (Olac Ltd, Bicester) were used in the two experiments. They were maintained throughout the experiment with unrestricted access to food (Breeders diet no. 3, Special Diet Services Ltd, Witham) and tap water. They were housed in a 12 h light/dark cycle, and all injections were administered between 0900 h and 1100 h. All the animals received a long-term, low-dose schedule of 1,2-dimethylhydrazine (Aldrich Chemical Co. Ltd, Poole) exposure, comprising 24 subcutaneous injections at one week intervals, of a dose of 15 mg (base) $kg^{-1}$ body weight. The animals were aged between 6 and 10 weeks at the start of the DMH treatment schedule and weighed between 150 g and 220 g. At the time of PMI experiments they were aged between 34 and 39 weeks and weighed between 350 g and 550 g. The animals were left for a period of between 3 and 5 weeks after the last DMH injection before subsequent experimentation. We adopt this procedure to allow complete recovery from any acute toxic effects of DMH.

### *High dose verapamil experiment (experiment 1)*

Twenty-six verapamil-treated animals all received verapamil (Abbott Laboratories, Queensbourough) at a dose of 100 mg $kg^{-1}$ body weight, administered by intraperitoneal injection in a vehicle comprising ethanol 40% v/v in normal saline. Thirty-four control (i.e. verapamil-untreated) animals received an intraperitoneal injection of the same volume of the vehicle only. Vincristine (Oncovin, Eli Lilly Ltd, Basingstoke) was administered at one of the following doses; 0.5, 0.25, 0.1, 0.05, 0.01 mg $kg^{-1}$ body weight, by i.p. injection. The drugs were administered in the following sequence: verapamil was administered 2 h prior to vincristine, and the animals were killed 2 h after vincristine injection. Thus the animals received verapamil or vehicle alone for 4 h, and vincristine in combination for the last two hours. This experimental protocol generates 10 permutations of drug administration (5 vincristine doses given either with or without verapamil). The animals were assigned to one of these 10 groups by a method adopted to optimise the distribution of colonic tumours between the groups. The experiment was performed in small batches selected at random sequentially over a number of days, with dissection of the colons to maintain a running total of tumours in each group. This procedure allowed the allocation of animals on subsequent days to groups where fewer tumours were present. A target of 6 colonic tumours in each group was achieved in 8 of the 10 groups.

The animals were killed by cervical dislocation and autopsy was performed immediately. The small bowel was dissected free and fixed unopened for at least 10 h in Carnoy's fluid. The colon was dissected free with a margin

Correspondence: P. Ince.
Received 8 September 1987; and in revised form, 11 December 1987.

of anal skin, opened along its length, and pinned to a cork board prior to Carnoy fixation. The tissues were subsequently transferred to cellosolve. Following fixation the specimens were carefully examined for tumours. Transverse blocks of the bowel were taken through each tumour, and through non-neoplastic mucosa at two sites prone to tumour development, *viz.* 20 mm distal to the pylorus, and at the junction of the middle and distal thirds of the colon. All these blocks were processed routinely to paraffin wax; histological sections prepared at 4 $\mu$m were stained with haematoxylin and eosin prior to counting.

*Verapamil dose response experiment (experiment 2)*

Twenty-eight animals received verapamil at one of the following doses; 100, 50, 25, 10, and 5 mg $kg^{-1}$ body weight. All the animals received vincristine at a single dose of 0.1 mg $kg^{-1}$ body weight. Otherwise the experiment was performed exactly as above.

*Counting procedures and statistical analysis*

The proportion of mitotic figures showing escape from metaphase arrest in each section of tumour or normal mucosa was obtained using the method that we have previously described (Ince *et al.*, 1985). The histological sections were counted as follows: whole circumferential sections of non-neoplastic mucosa were counted for the low doses of vincristine, and half or one third circumferences for the higher dose groups where metaphase arrest was more complete and mitotic figures thus much more numerous. Total number of mitotic figures was obtained, together with the total of unequivocally normal post-metaphase figures. The morphology of prophase is difficult to define, and it was decided to exclude this phase of mitosis from the study, although late prophase figures may have been included in the overall total. Using the counting method described the number of all mitoses counted in each section was 100–200 for low dose groups and 200–300 for high dose groups. In the case of tumours, areas of viable neoplastic tissue were selected at hazard and similar counts made. The total mitoses counted for each tumour was 100–300. In the subsequent analysis the total mitoses are designated 'm' and total normal post-metaphase figures 'a'.

The ratio of post-metaphase figures to all mitoses is the Post-Metaphase Index (PMI = a/m). The relationship between the PMI, which is an index of the degree of escape from metaphase arrest, and the dose of vincristine was analysed using the computer program GLIM (Baker & Nelder, 1978). The PMI was transformed to the logit;

$$\text{logit PMI} = \log_e \frac{(1-\text{PMI})}{(\text{PMI})}$$

$$= \log_e \frac{(m-a)}{(a)}$$

We selected the logit transformation rather than a simple logarithmic transformation because of the nature of the observations. Thus for each observed mitotic figure there are two 'all or nothing' options *viz.* metaphase or post-metaphase. This yields data in the form of a biological assay with quantal responses for which one possible transformation is the logistic (Finney, 1978). We found that this transformation together with transformation of vincristine dose to $\log_{10}$ dose, was the most satisfactory in order to linearise our data. These transformed data were plotted and the slopes of the fitted lines calculated by the computer model using the method of maximum likelihood (McCullagh & Nelder, 1983). The data for small bowel and large bowel were analysed separately; at both sites there are two tissues represented comprising DMH-treated non-neoplastic mucosa, and tumour. These were designated Mucosa and Tumour respectively. Analysis of deviance was performed in experiment 1 to test for differences between the slopes and positions of the fitted lines. The *t*-test was used based on this analysis to test for significant differences between the tissues, the sites, and between verapamil-treated and untreated tissues.

### Results

*Experiment 1*

The distribution of tumours and animals bearing tumours among the experimental groups is shown in Table I. Altogether 125 tumours were identified, 71 in the colon and 54 in the small bowel. The same range of tumour morphology was observed as that seen previously in DMH-treated rats (Sunter *et al.*, 1978), but all the variants were analysed together.

The mean PMI for each combination of state and tissue at each dose of vincristine, with or without verapamil, together with the standard error is shown in Table II. The data show a progressive rise in metaphase escape (an increasing PMI) as the dose of vincristine administered decreases. This corresponds with our previous experience of this kind of data (Ince *et al.*, 1985; 1986). In 15 of the 20 pairs of data (verapamil-treated *vs.* verapamil-untreated) there is less metaphase escape (i.e. a lower PMI) in the verapamil treated tissues. The dose-response relationship both in the presence and absence of verapamil is not linear. In order to test for a statistically significant difference between these dose response curves the data were transformed to linearity using the transformations of logit PMI and $\log_{10}$ dose as previously described. Figure 1 shows the transformed data points for one combination of tissue and treatments, viz. colon – tumours – verapamil, together with the fitted line calculated by the computer model. The fitted lines for all the transformed data are shown in Figures 2 and 3. We have plotted the negative logit PMI simply because we prefer to consider the phenomenon in terms of increasing metaphase arrest plotted against increasing vincristine dose.

Lines were fitted for the four highest doses of vincristine only. This is because of the minimal degree of metaphase arrest present at the lowest dose (see Figure 1). The fitted lines representing verapamil treated tissue lie parallel to the untreated. At the lowest dose of vincristine this relationship changes because the degree of escape from metaphase arrest is virtually maximal at this dose. Thus the PMI for 0.01 mg $kg^{-1}$ vincristine is insufficiently different from the 'native' PMI for an enhancing effect to occur. The figures show that for both tumour tissue and non-neoplastic mucosa, in both sites, the fitted lines for the verapamil-treated tissues lie above their control counterparts. These differences are not marked and, when tested taking each pair individually, do not achieve statistical significance. However, the GLIM program was used to generate the following mathematical function which summarises the whole of the data:

$$\begin{aligned} -\text{logit PMI} = 11.50 &+ 0.39 \text{ if small bowel} \\ &- 5.36 \text{ if tumour} \\ &+ 0.36 \text{ if verapamil} \\ &+ (6.50 - 3.46 \text{ if tumour}) \times \log_{10} \text{dose} \end{aligned}$$

In addition the program generates standard errors of these parameters allowing simple significance testing as follows:

| parameter | | s.e. | df | *t* value | |
|---|---|---|---|---|---|
| small bowel | 0.39 | 0.15 | 26 | $t = 2.53$ | $P < 0.05$ |
| tumour | −5.36 | 1.33 | 26 | $t = 4.02$ | $P < 0.001$ |
| tumour (slope) | −3.46 | 1.08 | 26 | $t = 3.20$ | $P < 0.01$ |
| verapamil | 0.36 | 0.16 | 26 | $t = 2.21$ | $P < 0.05$ |

This analysis uses the whole of the data together and shows significant reduction in vincristine resistance in the

**Table I** Distribution of intestinal tumours and of tumour bearing animals in experiment 1 by anatomical site, and verapamil and vincristine dosage group

| Anatomical site | Verapamil | a. No. of animals b. No. of animals with tumours c. No. of tumours | | | | | | | | | | | | | | | | | |
|---|---|---|---|---|---|---|---|---|---|---|---|---|---|---|---|---|---|---|---|
| | | *Vincristine dose* (*mg kg*$^{-1}$ *body weight*) | | | | | | | | | | | | | | | | | |
| | | *0.5* | | | *0.25* | | | *0.1* | | | *0.05* | | | *0.1* | | | (*Total*) | | |
| | | *a* | *b* | *c* | *a* | *b* | *c* | *a* | *b* | *c* | *a* | *b* | *c* | *a* | *b* | *c* | *a* | *b* | *c* |
| Colon | treated | 6 | 5 | 6 | 3 | 3 | 9 | 6 | 4 | 9 | 5 | 2 | 6 | 9 | 4 | 5 | 29 | 18 | 35 |
| | untreated | 4 | 4 | 8 | 6 | 4 | 7 | 8 | 6 | 8 | 7 | 5 | 10 | 9 | 3 | 3 | 34 | 22 | 36 |
| Small intestine | treated | 6 | 3 | 5 | 3 | 2 | 3 | 6 | 3 | 4 | 5 | 2 | 2 | 9 | 6 | 9 | 29 | 16 | 23 |
| | untreated | 4 | 2 | 3 | 6 | 4 | 6 | 8 | 5 | 8 | 7 | 4 | 7 | 9 | 5 | 7 | 34 | 20 | 31 |

**Table II** Effect of vincristine dose on the PMI% in the presence or absence of verapamil in each site/tissue combination. Standard errors are included in parenthesis and were calculated by the method of Snedecor and Cochran (1971)

| | *PMI%* | | | | | | | |
|---|---|---|---|---|---|---|---|---|
| | *Colon* | | | | *Small intestine* | | | |
| | *tumour* | | *mucosa* | | *tumour* | | *mucosa* | |
| *Vincristine dose* (*mg kg*$^{-1}$) | *treated* | *untreated* | *treated* | *untreated* | *treated* | *untreated* | *treated* | *untreated* |
| 0.0 | 16.0 (1.2) | | 15.0 (1.7) | | 16.8 (2.5) | | 18.5 (2.1) | |
| 0.1 | 11.5 (1.3) | 15.5 (2.4) | 9.5 (1.8) | 11.3 (1.6) | 14.2 (1.9) | 13.8 (2.1) | 8.1 (2.0) | 7.8 (3.1) |
| 0.05 | 8.2 (1.4) | 10.5 (1.5) | 4.4 (1.5) | 4.5 (2.2) | 3.8 (0.2) | 6.5 (2.5) | 3.3 (1.5) | 2.3 (1.4) |
| 0.10 | 2.9 (0.9) | 3.1 (1.2) | 0.3 (0.1) | 0.9 (0.5) | 1.1 (0.4) | 5.1 (0.1) | 0.2 (0.1) | 0.4 (0.3 |
| 0.25 | 0.8 (0.3) | 1.7 (0.9) | 0.2 (0.1) | 0 | 0.1 (0.1) | 1.0 (0.5) | 0 | 0.1 (0.1) |
| 0.5 | 0.2 (0.1) | 0.5 (0.2) | 0 | 0 | 0.5 (0.4) | 0.2 (0.1) | 0 | 0 |

**Figure 1** Experiment 1 colon – tumours – verapamil. The data points and fitted line for one combination of tissue and treatment. The circles and arrows represent PMI values of 0 (i.e. logit PMI = −∞). The lowest dose of vincristine (0.01) shows PMI values approaching that of a group of verapamil only controls. This latter group has been transposed to the right hand side for clarity. The dotted section of the line represents an extrapolation from the line fitted between 0.05 and 0.5 ($\log_{10}$ mg kg$^{-1}$ body wt.) at lower doses of VCR. It clearly does not correspond to the data over this dose range.

**Figure 2** Experiment 1 – colon. All 4 fitted lines for verapamil treated (-----) and control (——) tissues. The higher the position of a line the greater the degree of metaphase arrest, i.e. the greater the sensitivity to vincristine.

presence of verapamil at an administered dose of 100 mg kg$^{-1}$ ($P<0.05$). In addition the data confirm our previously reported findings of relative vincristine resistance in the colon compared with the small bowel ($P<0.05$), and in tumour tissue compared with normal mucosa ($P<0.001$).

*Experiment 2*

The distribution of intestinal tumours between the verapamil dose groups is shown in Table III. A total of 75 tumours were identified and a goal of 7 colonic tumours per dose group was achieved in 3 of the 5 groups. As previously described all morphological groups of tumours were analysed together.

**Figure 3** Experiment 1 – small intestine. As **Figure 2**, verapamil-treated (-----) and control (——) tissues.

The mean PMI% for tumours at each dose of verapamil is shown in Table IV. At this dose of vincristine (0.1 mg $kg^{-1}$) there is only minimal escape from metaphase arrest in non-neoplastic mucosa and this tissue was excluded from the analysis. The mean PMI% for the tumour tissues in this experiment at the verapamil dose given in the first experiment are 1.9 in the colon and 1.2 in the small bowel, and they are not significantly different from the values for the same vincristine dose (verapamil-treated) in experiment 1 of colon 2.9, and small bowel 1.1. We examined the data using the mathematical transformation as described above to look for a significant decrease in escape from metaphase arrest with increasing dose of verapamil. This is illustrated in Figures 4 and 5. The individual data points are included as well as the fitted lines. The fitted lines illustrated are curved because the abscissa plots the untransformed PMI%. The fitted line for colonic tumours shows the expected effect, that for small bowel tumours shows an apparent reversal of this trend. The equations describing these lines are as follows:

colon logit PMI $= -2.98 - 0.74 \times \log$ dose small bowel logit PMI $= -4.36 + 0.23 \times \log$ dose

Using analysis of deviance standard errors for the slopes of the lines were calculated as follows:

| parameter | | s.e. | df | *t* value | |
|---|---|---|---|---|---|
| slope-colon | −0.74 | −0.35 | 29 | 2.09 | $P<0.05$ |
| slope-small bowel | 0.23 | −0.33 | 28 | 0.71 | NS |

Our analysis shows a significant slope of the fitted line for the colonic tumours but not for small bowel tumours. Thus we have demonstrated a slight increase in the sensitivity to vincristine in colonic tumours in the presence of increasing doses of verapamil. The failure to demonstrate an effect in small bowel tumours in this experiment may be due to the rather small amount of data available but, in view of the negative value for the slope, it probably reflects the lower level of vincristine resistance present in this tissue.

**Figure 4** Experiment 2 – colon. Data points and fitted lines for tumour tissue. The ordinate shows the untransformed PMI thus the fitted line appears curved. The negative slope of the line is significantly different from 0 ($P<0.05$).

**Figure 5** Experiment 2 – small intestine. Data points and fitted line for tumour tissue. The ordinate shows the untransformed PMI thus the fitted line appears curved. The positive slope is not significantly different from 0 ($P>0.05$).

### Discussion

We have previously shown that the PMI offers a morphological means of measuring the degree of resistance to the stathmokinetic effect of vincristine within a tissue (Ince *et al.*, 1985). This method has the advantage over biochemical methods of estimating parameters related to drug resistance in intact tissues in that the measurements made relate exclusively to the epithelial tissue. Our previous work has also shown that the technique is sensitive enough *in vitro* to show differences between the resistance of tumour tissues when treated with a sensitising agent as compared

**Table III** Distribution of intestinal tumours and tumour bearing animals in experiment 2 by anatomical site and verapamil dosage group. All animals received vincristine at 0.1 mg $kg^{-1}$

*a. No of animals*
*b. No. of animals with tumours*
*c. No. of tumours*

| *Anatomical site* | *Verapamil dose (mg kg⁻¹ body weight)* 100 | | | 50 | | | 25 | | | 10 | | | 5 | | | (Total) | | |
|---|---|---|---|---|---|---|---|---|---|---|---|---|---|---|---|---|---|---|
| | *a* | *b* | *c* | *a* | *b* | *c* | *a* | *b* | *c* | *a* | *b* | *c* | *a* | *b* | *c* | *a* | *b* | *c* |
| Colon | 5 | 3 | 10 | 6 | 3 | 10 | 5 | 4 | 5 | 6 | 3 | 9 | 6 | 3 | 4 | 28 | 16 | 38 |
| Small intestine | 5 | 2 | 10 | 6 | 4 | 8 | 5 | 3 | 9 | 6 | 4 | 7 | 6 | 3 | 6 | 28 | 16 | 29 |

Table IV Effect of verapamil on the PMI% in tumour tissue at each site at vincristine dose of $0.1\,mg\,kg^{-1}$. Standard errors are included in parenthesis and were calculated by the method of Snedecor and Cochran (1971)

| | PMI% | | | | |
|---|---|---|---|---|---|
| | Verapamil dose ($mg\,kg^{-1}$) | | | | |
| Anatomical site | 100 | 50 | 25 | 10 | 5 |
| Colon | 1.9 | 0.8 | 2.5 | 2.3 | 3.4 |
| | (0.9) | (0.7) | (1.4) | (2.9) | (1.5) |
| Small intestine | 1.2 | 3.0 | 1.4 | 1.4 | 1.8 |
| | (1.0) | (2.7) | (0.7) | (1.0) | (0.8) |

with control untreated tissue (Ince *et al.*, 1986). *In vivo* we have shown differences between normal and neoplastic tissues and between different regional types of epithelial tissues in the gut.

The first experiment described here shows that using the PMI it is possible to show a sensitising effect of verapamil ($P<0.05$). It is apparent from the data that this effect is small. We have shown a similar degree of tumour cell resistance compared with normal mucosa to that which we have previously reported. Verapamil does not reduce the resistance of tumour tissue to that of normal mucosa. The pharmacokinetics of verapamil are of importance in the interpretation of the experiment. The administered dose of $100\,mg\,kg^{-1}$ resulted in the death of 3 animals in the treated group (~LD10). This is a high dose and was selected to ensure the maximum tissue levels compatible with an adequate survival to the end of the experiment. In clinical therapy the highest achievable dose of verapamil is roughly equivalent to $5–10\,mg\,kg^{-1}$. In the second experiment reported here this dose range is associated with minimal enhancement of vincristine sensitivity. Thus it would seem that an effective clinical application of modifiers will require compounds active at lower doses.

The data presented here show no distinction between the effect of verapamil on normal compared with neoplastic tissues. This may reflect a similar sensitising effect of verapamil on both tissues, but this remains to be established. The standard errors of the means are large, particularly so in experiment 2, and larger numbers of tumours and animals would be needed to resolve this problem. We are currently investigating this aspect of the resistance of normal mucosa using our explant organ culture system. In this context the high level of mRNA for the p-glycoprotein reported in both tumourous and normal human colonic tissues is of interest (Fojo *et al.*, 1987). This finding can be interpreted as evidence of a naturally occurring cellular mechanism to protect the colon, amongst other tissues, from the effects of plant toxins. Taken further the theory can be used to account for both the enhanced resistance of MDR cells, and the resistance of spontaneous tumours on the basis of increased activity of this normal mechanism. Many tissues show low levels of p-glycoprotein mRNA including haemopoetic tissue. If modulators can only act on tissues possessing the p-glycoprotein then the bone marrow should be spared the effects of enhanced sensitivity. The PMI technique provides an opportunity to investigate this therapeutically important point.

In terms of the investigation of the relationship between the innate resistance of primary solid tumours and the acquired MDR phenotype we have shown both here *in vivo*, and previously *in vitro* using human tissue (Ince *et al.*, 1986), that the innate resistance to vincristine of colonic cancer shares a pharmacological property with MDR, *viz.* modulation of resistance by verapamil. Other evidence is required to elucidate this link. Studies using monoclonal antisera to the MDR-related p-glycoprotein in patients relapsing from acute nonlymphoblastic leukaemia have demonstrated resistant tumour cells containing large amounts of antigen (Kartner *et al.*, 1985, Ma *et al.*, 1987). Blot analysis of a wide range of human tissues, both normal and malignant, has shown higher levels of cytoplasmic m-RNA coding for p-glycoprotein in adrenal, renal and colonic tissues compared with others (Fojo *et al.*, 1987). Biochemical studies have shown decreased drug accumulation in human tumour cells lines derived from haematological malignancies (Ferguson & Cass, 1985) and carcinoma tissue (Fojo *et al.*, 1985). However, there is no evidence concerning the biochemical activity of the p-glycoprotein in native colonic tumour cells or in normal colonic mucosa. We are currently investigating the accumulation, localisation and efflux of tritiated alkaloids in normal colonic mucosa to compare these properties with those described in MDR.

Histological sections were prepared by Mrs K. Elliot. Graphs were prepared by Mr W. Robinson, and secretarial assistance provided by Miss E. Wark. This study was supported by a grant from the North of England Cancer Research Campaign.

### References

BAKER, R.J. & NELDER, J.A. (1978). The GLIM System (Release 3) Manual. *Numerical Algorithms Group for the Royal Statistical Society*.

FERGUSON, P.J. & CASS, C.E. (1985). Differential cellular retention of vincristine and vinblastine by cultured human promyelocytic leukaemia cells HL60/CI Cells: The basis of differential toxicity. *Cancer Res.*, **45**, 5480.

FINNEY, D.J. (1978). *Statistical Method in Biological Assay*. 3rd ed. Griffin & Co. Ltd.: London p. 358.

FOJO, A., AKIYAMA, M.M. & PASTAN, I. (1985). Reduced drug accumulation in drug-resistant human carcinoma cell lines. *Cancer Res.*, **45**, 3002.

FOJO, A.T., UEDA, K., SLAMON, D.J., POPLACK, D.G., GOTTESMAN, M.M. & PASTAN, I. (1987). Expression of a multidrug-resistance gene in human tumours and tissues. *Proc. Natl. Acad. Sci.*, **84**, 265.

INCE, P., APPLETON, D.R., FINNEY, K.J., SUNTER, J.P. & WATSON, A.J. (1986). Verapamil increases the sensitivity of primary human colorectal carcinoma tissue to vincristine. *Br. J. Cancer*, **53**, 137.

INCE, P., FINNEY, K.J., APPLETON, D.R., SUNTER, J.P. & WATSON, A.J. (1985). Demonstration of vincristine resistance in primary intestinal neoplasms in the rat by the 'Post-metaphase Index'. *Br. J. Cancer*, **52**, 599.

KARTNER, N., EVERNDEN-PORELLE, D., BRADLEY, G. & LING, V. (1985). Detection of P-glycoprotein in multidrug resistant cell lines by monoclonal antibodies. *Nature*, **316**, 820.

LING, V., KARTNER, N., SUDO, T., SMINOVITCH, L. & RIORDAN, J.R. (1983). Multi-resistance phenotype in Chinese hamster ovary cells. *Cancer Treat Rep.*, **67**, 869.

MA, D.D.F., SCURR, R.D., DAVEY, R.A. & 5 others (1987). Detection of multidrug resistant phenotype in acute non-lymphoblastic leukaemia. *Lancet*, **i**, 135.

McCULLAGH, P. & NELDER, J.A. (1983). *Generalised Linear Models*. Chapman and Hall: London.

SKOVSGAARD, T., DANØ, K. & NISSEN, N.I. (1984). Chemosensitizers counteracting acquired resistance to anthracyclines and vinca alkaloids *in vivo*. A new treatment principle. *Cancer Reviews* II Suppl. A. 63.

SNEDECOR, G.W. & COCHRAN, W.G. (1971). *Statistical Methods*. 6th ed. Iowa State University Press: Ames Iowa p. 511.

SUNTER, J.P., APPLETON, D.R., WRIGHT, N.A. & WATSON, A.J. (1978). Pathological features of the colonic tumours induced in rats by the administration of 1,2-Dimethylhydrazine. *Virchows Arch.* (*Cell Pathol.*), **29**, 211.

TSURUO, T. (1983). Reversal of acquired resistance to vinca alkaloids and anthracycline antibiotics. *Cancer Treat Rep.*, **67**, 889.